# Buckling Behavior of Substrate Supported Graphene Sheets

**DOI:** 10.3390/ma9010032

**Published:** 2016-01-07

**Authors:** Kuijian Yang, Yuli Chen, Fei Pan, Shengtao Wang, Yong Ma, Qijun Liu

**Affiliations:** 1 Institute of Solid Mechanics, International Research Institute for Multidisciplinary Science, Beihang University, Beijing 100191, China; yangkj@buaa.edu.cn (K.Y.); f_pan@buaa.edu.cn (F.P.); wangst@buaa.edu.cn (S.W.); mayong@buaa.edu.cn (Y.M.); 2 Department of Aerospace Engineering, University of Illinois, Champaign, IL 61801, USA; qliu38@illinois.edu

**Keywords:** graphene, buckling, molecular mechanics, energy method, nano-electromechanical system

## Abstract

The buckling of graphene sheets on substrates can significantly degrade their performance in materials and devices. Therefore, a systematic investigation on the buckling behavior of monolayer graphene sheet/substrate systems is carried out in this paper by both molecular mechanics simulations and theoretical analysis. From 70 simulation cases of simple-supported graphene sheets with different sizes under uniaxial compression, two different buckling modes are investigated and revealed to be dominated by the graphene size. Especially, for graphene sheets with length larger than 3 nm and width larger than 1.1 nm, the buckling mode depends only on the length/width ratio. Besides, it is revealed that the existence of graphene substrate can increase the critical buckling stress and strain to 4.39 N/m and 1.58%, respectively, which are about 10 times those for free-standing graphene sheets. Moreover, for graphene sheets with common size (longer than 20 nm), both theoretical and simulation results show that the critical buckling stress and strain are dominated only by the adhesive interactions with substrate and independent of the graphene size. Results in this work provide valuable insight and guidelines for the design and application of graphene-derived materials and nano-electromechanical systems.

## 1. Introduction

Graphene has drawn widespread attention due to their excellent properties in electricity, thermology, optics and mechanics, and thus plays an important role in nano-materials and nano-devices [1,2,3,4,5,6,7,8]. However, as a two-dimensional nano material, graphene sheets are easily wrinkled and buckled under compression, which weakens their mechanical properties significantly. Therefore, in order to make better use of the excellent properties of graphene and improve the properties of graphene-based materials and devices, a comprehensive understanding on the buckling behaviors of graphene sheets is in great demand.

In recent years, experimental and theoretical studies have been carried out to investigate the buckling behavior of graphene sheets [9,10,11,12,13,14,15,16,17,18,19,20,21,22]. Most of them focused on free-standing graphene sheets, and only a few reported the buckling of substrate-supported graphene sheets. In practical applications, graphene sheets are normally used with substrate materials [23,24,25], and it is therefore of great importance to study the buckling behavior of substrate-supported graphene sheets. Anjomshoa *et al.* [19] and Wang *et al.* [20] investigated the compression behavior of the graphene embedded in polymer matrix. Jiang *et al.* [21] investigated the buckling of monolayer graphene on polyethylene terephthalate (PET) under compression using *in-situ* Raman spectroscopy and atomic force microscopy. They reported a critical compressive strain of around 0.7%. By assuming a simple failure mode, Wilber [22] formulated a continuum model of a graphene sheet supported by a flat rigid substrate and studied the effects of boundary conditions, substrate, and sheet length on the buckling of the sheet based on bifurcation theory. He obtained a theoretical buckling stress much lower than the simulation results, which mainly results from the inaccurate assumption of buckling mode.

However, in previous studies, the buckling modes of substrate-supported graphene sheets are rarely reported. The buckling mode determines the assumption of theoretical model, and it thus affects the critical buckling stress and critical buckling strain significantly. In this paper, the all-atom simulations are carried out to investigate the buckling modes as well as the buckling stress and strain of a monolayer graphene sheet subjected to a uniaxial compressive load on a flat rigid substrate. The effects of sheet size are also studied, and the critical condition for different buckling modes is obtained. Furthermore, the theoretical analysis is carried out based on the energy method to investigate the mechanism of buckling behavior and to reveal the substrate effect on the buckling stress and strain.

## 2. Atomistic Simulation

In this section, the graphene substrate is taken as an example to simulate the buckling behaviors of graphene sheets. The simulation method and the qualitative conclusion are also applicable to the graphene systems with other substrate materials.

### 2.1. Model and Method

**Figure 1:**
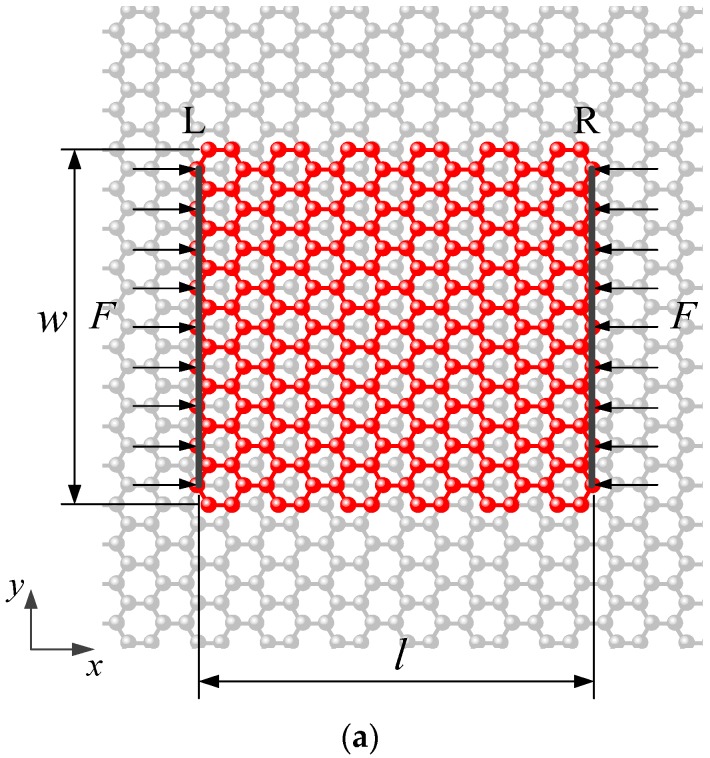

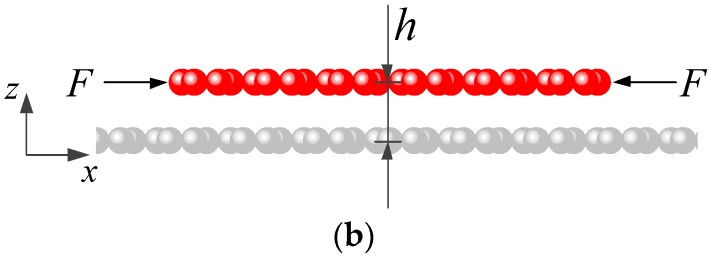
Sketch of a graphene sheet subjected to the compressive force *F* on an infinite large graphene substrate: (**a**) top view; and (**b**) side view.

Figure 1 presents the simulation model of a rectangular monolayer graphene sheet with the length *l* and the width *w* on a rigid graphene substrate. The substrate is modeled as a rigid body because it is much thicker than the graphene and thus the deformation is constrained by the lower-layer material. The compressive loads are applied on the zigzag edges of the graphene sheet, as shown in Figure 1. Both zigzag edges, denoted by L and R, are simply supported, which means the out-of-plane displacements (along *z*-direction) of all the atoms on edges L and R are restrained. Besides, along the armchair-edge direction (*x*-direction), the displacements of atoms on edge L (or R) are uniform, and their displacements along the zigzag-edge direction (*y*-direction) are unconfined. When loading, edges L and R move inside along the loading direction, and their displacements are the same in magnitude but opposite in direction.

The atomistic simulation on the deformation of graphene on rigid substrate under compressive load is carried out by the atomic-scale finite element method (AFEM) [26,27,28], which is essentially an effective and accurate molecular mechanics method based on the minimum potential energy theorem. The AFEM code has exactly the same formal structure as continuum finite element methods, and therefore can seamlessly be combined with the pre- and post- process codes of finite element software to apply boundary conditions and output atom positions and forces. Hence it has been widely used to solve nanoscale problems, especially for the carbon atom systems [26,27,29,30,31].

The second-generation Tersoff–Brenner potential [32] is adopted in the simulation to describe the covalent C–C bonds, and the initial length of C–C bond is 0.1425 nm. The van der Waals interactions between graphene sheet and substrate are described by the Lennard–Jones 6–12 potential [33], in which the potential energy between a carbon atom and a substrate atom is:

(1) $$V\left(d\right)=4\varepsilon_{LJ}{\left[\left(\frac{\sigma_{LJ}}{d}\right)\right]}^{12}-{\left[\left(\frac{\sigma_{LJ}}{d}\right)\right]}^{6}$$

where *d* is the distance between the two atoms, $\varepsilon_{LJ}$ is the energy at the equilibrium distance $\text{d}=\sqrt[{6}]{2}\sigma_{LJ}$, and $\sigma_{LJ}$ is the characteristic distance, which has been proved to be the equilibrium distance between an atom and an infinite plane substrate [34]. For graphene substrate, $\varepsilon_{LJ}=2.39\;\times \;10^{-3}\;eV$ and $\sigma_{LJ}=0.34\;nm$ [29,35].

The initial configuration of graphene is generated as an ideal perfect two-dimensional sheet with hexagonal lattices in the plane. The initial distance between the graphene sheet and the substrate is 0.34 nm. Before compression, the graphene is relaxed to reach its minimum energy state, which is the initial equilibrium state. Then, the compressive loadings are applied to edges L and R by controlling the displacements. The deformation and buckling behavior of the substrate-supported graphene sheets are investigated by recording the atom coordinates at each displacement increment, and the total compressive force *F* is obtained by summing up the loading-direction force components of all the atoms on either of the loading edges.

### 2.2. Buckling and Post-Buckling Process

**Figure 2:**
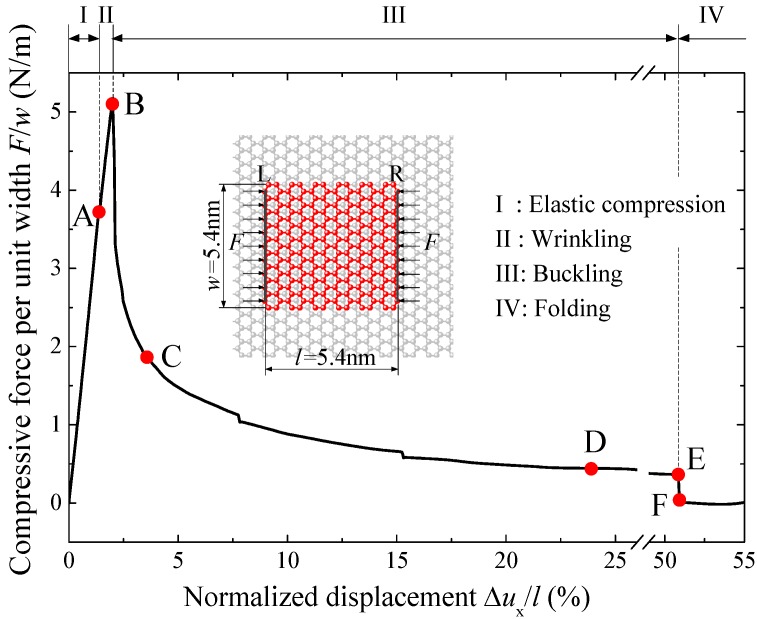
The compressive stress *versus* normalized displacement of the monolayer graphene sheet supported by a graphene substrate.

Figure 2 presents the relation between compressive force and normalized displacement of the graphene sheet with length *l* = 5.4 nm and width *w* = 5.4 nm. In Figure 2, the compressive force *F* is divided by the sheet width *w*, which is essentially equivalent to the compressive stress of the graphene sheet. The normalized displacement is defined as the relative displacement Δ*u_x_* divided by the total sheet length *l*, which is essentially the contraction ratio of the graphene sheet and corresponds to the compressive strain of the graphene sheets before buckling. Here, $\Delta u_{x}=\;u_{x}^{\text{L}}$ −$\;u_{x}^{\text{R}}$ is the loading displacement of edge L relative to that of edge R, which is a scalar component of displacement vector.

**Figure 3:**
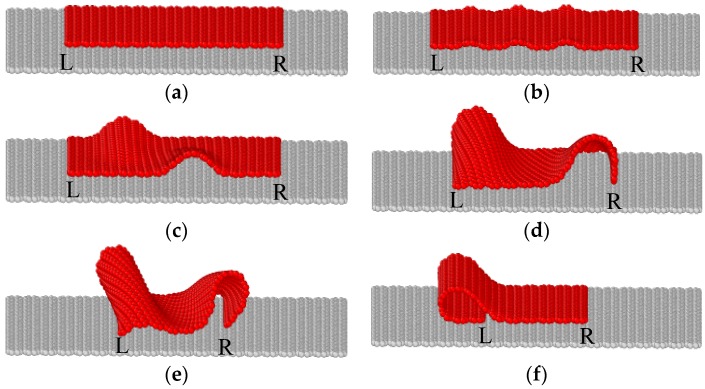
Configurations of the graphene sheet at points A–F of the curve in Figure 2. (**a**) Δ*u_x_*/*l* = 1.37%; (**b**) Δ*u_x_*/*l* = 2.00%; (**c**) Δ*u_x_*/*l* = 3.71%; (**d**) Δ*u_x_*/*l* = 24.09%; (**e**) Δ*u_x_*/*l* = 50.89%; (**f**) Δ*u_x_*/*l* = 50.93%.

Figure 3 shows the deformed configurations of the graphene sheet at points A–F of the curve in Figure 2. According to the configurations, the curve can be divided into four stages: elastic compression, wrinkling, buckling and folding. Under compressive loading, the graphene first has a linear elastic in-plane deformation until point A. In this stage, both stress and strain of the graphene sheet increases linearly with the contraction, and the slope of the curve in Figure 2 is very high, indicating the extremely high modulus of the graphene. After Point A, the slope of the curve becomes a little lower because the graphene sheet wrinkles slightly on the substrate, and the compressive force keeps increasing until reaching the peak point B. This stage is very short and can only be observed in the graphene sheets with a certain range of length and width. At point B, the three small wrinkles reduces to two larger anti-symmetric waves, as shown in Figure 3c, and the force drops suddenly, which indicates the buckling of the graphene sheet. After that, with the increase of compressive displacement, the two anti-symmetric waves becomes larger and moves outside to the loading edges (Figure 3d,e), and finally converges to one edge suddenly, as shown in Figure 3f. After this sudden change, the sheet is folded and the compressive load drops almost to zero, as shown in Figure 2.

**Figure 4:**
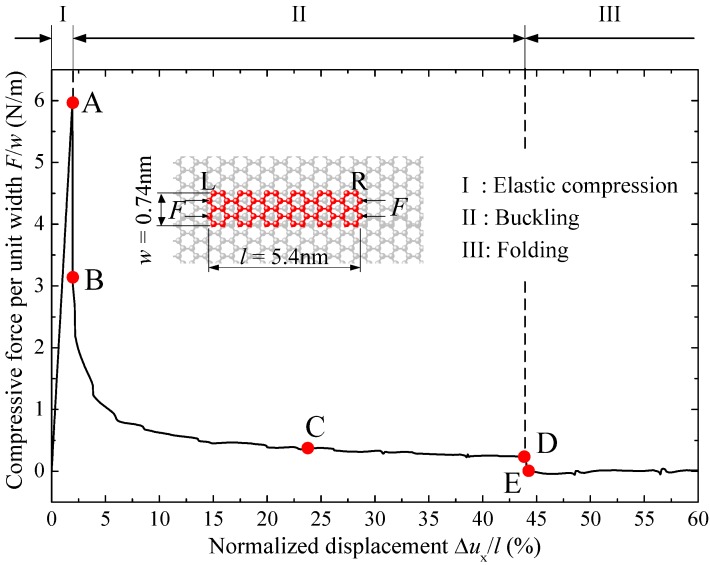
The compressive stress *versus* normalized displacement of the narrow monolayer graphene sheet supported by a graphene substrate.

**Figure 5:**
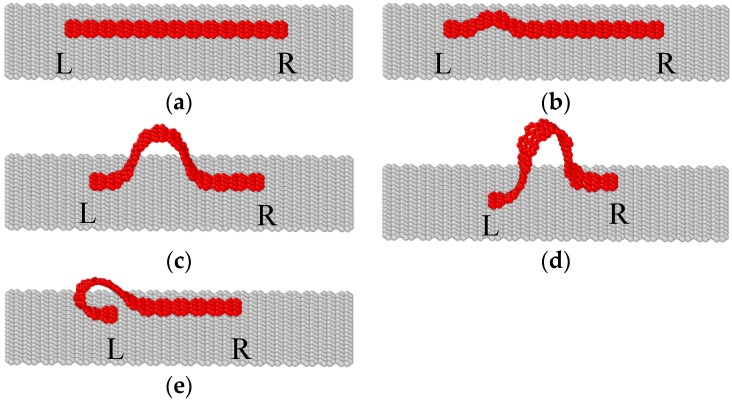
Configurations of the graphene sheet at points A–E of the curve in Figure 4. (**a**) Δ*u_x_*/*l* = 1.85%; (**b**) Δ*u_x_*/*l* = 2.59%; (**c**) Δ*u_x_*/*l* = 23.72%; (**d**) Δ*u_x_*/*l* = 44.00%; (**e**) Δ*u_x_*/*l* = 44.11%.

It is interesting to find that not all graphene sheets buckle following above mode. Figure 4 presents the deformation and buckling process of a narrow graphene sheet on the substrate. The length *l* of the narrow graphene sheet is 5.4 nm, same as the case of Figure 2, but the width *w* is 0.74 nm, much smaller than that of the sheet in Figure 2. Different from Figure 2, the narrow graphene sheet displays only three stages in Figure 4: elastic compression, buckling and folding, and the deformed configurations at points A–E of the curve are presented in Figure 5.

In the first stage, both stress and strain of the graphene sheet increases linearly with the compression, and the slope of the curve in Figure 4 is exactly same as that in Figure 2, because the elastic modulus of graphene sheets is a constant and independent of the shape. With the increase of the compressive displacement, the graphene changes directly from flat to buckled configurations, without any wrinkles before buckling. After buckling, only one wave appears. Then, the sheet twists slightly due to the relative transverse movement of the two loading edges, as illustrated in Figure 5d. If the loading edges keep moving inside, the graphene sheet becomes folded suddenly, as shown in Figure 5e.

### 2.3. Buckling Mode

**Figure 6:**
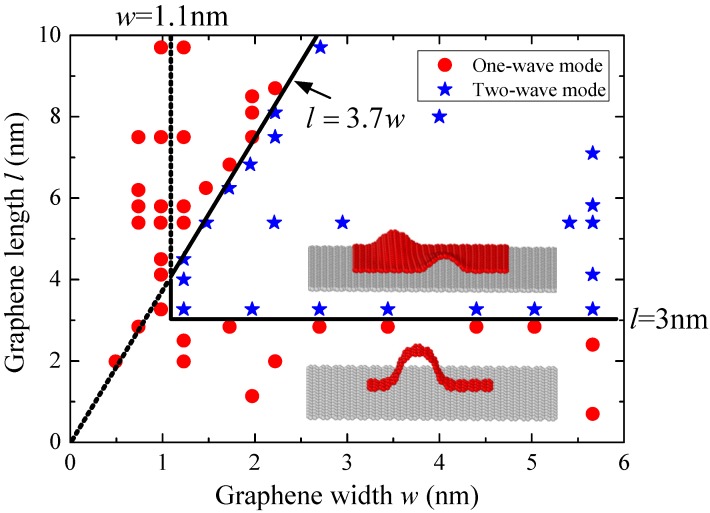
The buckling modes of monolayer graphene sheets with different sizes under uniaxial compression.

From Figure 3 and Figure 5, it can be investigated that the size of graphene sheet can affect the buckling mode. In order to have a deep insight into the size effect on the buckling mode of substrate-supported graphene sheets, 70 models of graphene sheets with different sizes are established and simulated, and the buckling modes are presented in Figure 6.

For the substrate-supported graphene sheet under uniaxial compression, if the sheet length less than 3 nm or the sheet width less than 1.1 nm, the only possible buckling mode is the one-wave mode similar to that presented in Figure 5. In this case, only one through wave appears when the sheet buckles, because the sheet with width less than 1.1 nm is so narrow that only through waves can be formed, and the sheet with length less than 3 nm is too short to form two or more waves.

Otherwise, if the sheet length is larger than 3 nm and the sheet width is larger than 1.1 nm, the buckling mode depends on the length/width ratio of the graphene sheet. The buckling of graphene sheets with the length/width ratio *l*/*w* larger than 3.7 still follows the one-wave mode. However, for the graphene sheets with the length/width ratio *l*/*w* less than 3.7, the buckling presents two-wave mode, similar to that illustrated in Figure 3. In this case, wrinkles are likely to appear before buckling and two anti-symmetric waves can be investigated after buckling.

### 2.4. The Critical Buckling Stress and Strain

The size of graphene sheet can also affect the critical buckling stress and strain, which can be obtained from the peak point of the curves demonstrated in Figure 2 and Figure 4.

**Figure 7:**
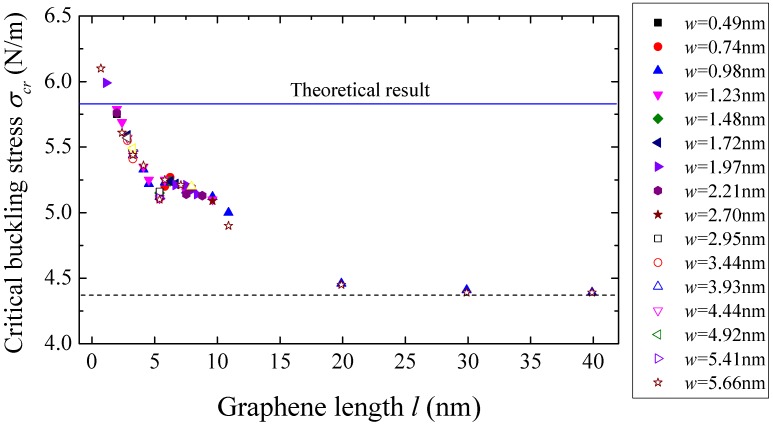
The critical buckling stress *versus* length of the monolayer graphene sheets with different sizes on a graphene substrate.

From the simulation results, the critical buckling stress, *i.e.*, the critical buckling force per unit width, decreases with increasing sheet length and approaches to 4.39 N/m, as shown in Figure 7. Especially, when the sheet length is larger than 20 nm, the critical buckling stress becomes a constant. This length independence property is consistent with the free-standing monolayer graphene sheet, but the constant buckling stress 4.39 N/m is about 10 times of the free-standing graphene sheets [12]. This difference results from the adhesion energy provided by the substrate. For free-standing graphene sheets, the buckling of graphene sheets is determined only by the strain energy, which is composed of the membrane energy and the bending energy. For the substrate-supported graphene sheets, the existence of adhesion energy suppresses the bending deformation and thus postpones the buckling.

**Figure 8:**
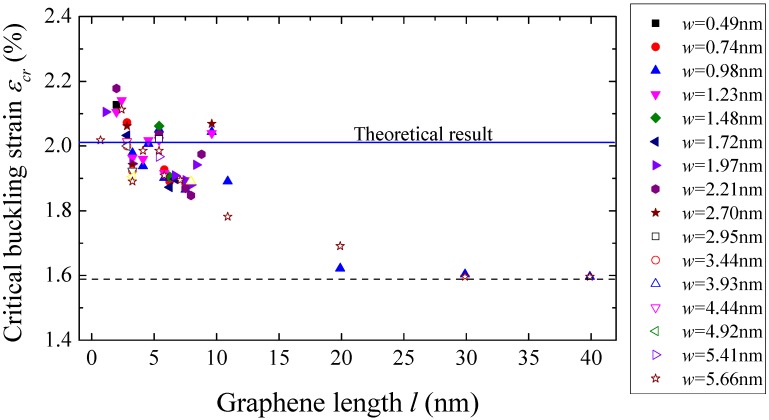
The critical buckling strain *versus* width of the monolayer graphene sheets with different sizes on a graphene substrate.

It is also found from the simulation results that the critical buckling strain is not sensitive to the graphene size, as shown in Figure 8. For the graphene sheet with length larger than 20 nm, the critical buckling strain is almost a constant, which is about 1.58%. This critical buckling strain is higher than the experimental result by Jiang *et al.* [21] because the graphene sheet is perfect in the simulation while it could have defects in experiments.

## 3. Theoretical Analysis

To obtain a better understanding on the buckling mechanism of the supported graphene sheets, the theoretical analysis is carried out by energy method [36,37].

The deformation and buckling behavior of the graphene sheets results from the competition between two energy terms [25,36,37,38]: the adhesion energy between graphene and substrate, which tries to keep the graphene sheet flat and attached completely to the substrate, and the strain energy stored in the graphene sheets due to the in-plane compressive deformation and the bending deformation. When the contraction ratio is small, the adhesion energy dominates the total energy of the graphene sheets, and the strain energy is relatively low, so the graphene can keep flat and completely conform to the substrate. With increasing compression, the strain energy becomes larger and larger, and the graphene tends to leave away from the substrate to reduce the membrane energy by bending deformation, and thus the sheet begins to wrinkle and/or buckle.

### 3.1. Energy Analysis

When the rectangular graphene sheet in Figure 1 is subjected to a uniaxial compression, denote the membrane strain of the sheet by ε*_m_* and the out-of-plane displacement by *u_z_*. Suppose the out-of-plane displacement depends only on *x* as *u_z_* = *A*·cos(π*x*/λ), in which *A* is the corrugation amplitude and λ is half of the wavelength. The membrane strain of graphene sheet can thus be obtained from von Karman nonlinear plate theory [39]:

(2) $$\varepsilon_{m}=\;-\frac{\Delta u_{x}}{l}+\frac{1}{4}{\left(\frac{\pi A}{\lambda }\right)}^{2}$$

The elastic strain energy density of graphene is

(3) $$U_{m}=\;\frac{1}{2}Et\varepsilon_{m}^{2}=\;\frac{1}{2}Et{\left(-\frac{\Delta u_{x}}{l}+\;\frac{\pi^{2}A^{2}}{4\lambda^{2}}\right)}^{2}$$

where *Et* = 289 N/m is the surface Young’s modulus, obtained due to the linearity range of compressive force curve (see Figure 2).

The bending energy density is:

(4) $$U_{b}=\;\frac{1}{2\lambda }\frac{D}{2}\int_{S}{\left(\frac{{\text{d}}^{2}u_{z}}{\text{d}x\text{d}s}\right)}^{2}\text{ds}=\frac{D}{4\lambda }\int_{-\lambda }^{\lambda }\frac{{\left(\frac{A\pi^{2}}{\lambda^{2}}\;\text{cos}\left(\frac{\pi }{\lambda }x\right)\right)}^{2}}{\sqrt{1+{\left(\frac{A\pi }{\lambda }\;\text{sin}\left(\frac{\pi }{\lambda }x\right)\right)}^{2}}}\text{d}x$$

where *s* is the curve segment along the free edge of graphene sheet, and D = 0.238 nN·nm [40] is the bending modulus of graphene.

The adhesion energy density between graphene and substrate can be obtained from the integral of Lennard-Jones potential over the area [35]:

(5) $$U_{ad}=\;\frac{1}{2\lambda }\int_{s}\rho_{c}\int_{A_{s}}\rho_{s}V\left(d\right)dA_{s}\text{ds}$$

where ρ_c_ and ρ_s_ are the atom area density of graphene and substrate, respectively, and *A_s_* is the infinite substrate area. For graphene substrate, $\rho_{s}=\rho_{c}=4/\left(3\sqrt{3}l_{c–c}^{2}\right)$ and $l_{c–c}^{2}=0.1421\;\text{nm}$ is the equilibrium bond length of graphene. Using $\phi_{s}=1.2\pi \rho_{c}\rho_{s}\varepsilon_{LJ}\sigma_{LJ}^{2}$ to denote the adhesion energy density of graphene at the characteristic distance $d=\sigma_{LJ}$, Equation (5) can be rewritten as:

(6) $$U_{ad}=\;\frac{1}{2\lambda }\int_{-\lambda }^{\lambda }\frac{5\phi_{s}}{3}\left(\frac{2\sigma_{LJ}^{10}}{5{\left(\sigma_{LJ}+A\text{cos}\left(\frac{\pi }{\lambda }x\right)\right)}^{10}}\;-\frac{\sigma_{LJ}^{4}}{{\left(\sigma_{LJ}+A\text{cos}\left(\frac{\pi }{\lambda }x\right)\right)}^{4}}\right)\sqrt{1+{\left(\frac{A\pi }{\lambda }\;\text{sin}\left(\frac{\pi }{\lambda }x\right)\right)}^{2}}\text{d}x$$

From Equations (3), (4) and (6), the total energy density of graphene is:

(7) $$U_{T}=\;U_{m}\left(\frac{\Delta u_{x}}{l},A,\lambda \right)+\;U_{b}\left(A,\lambda \right)+\;U_{ad}\left(A,\lambda \right)$$

Obviously, the total energy density *U_T_* is a function of contraction ratio $\Delta u_{x}/l$, amplitude *A* and half wavelength λ. For each given contraction ratio $\Delta u_{x}/l$, the equilibrium state can be obtained by minimizing the total energy:

(8) $$\frac{\partial U_{T}}{\partial \text{A}}=0\;and\;\frac{\partial U_{T}}{\partial \lambda }=0\;$$

### 3.2. Numerical Solution

The equation set in Equation (8) can be solved numerically. When the contraction ratio $\Delta u_{x}/l$ is small, the amplitude *A* = 0 is solved from Equation (8). With increasing $\Delta u_{x}/l$, at a critical point, the amplitude becomes larger than zero, and the contraction ratio at this point is the critical buckling strain $\varepsilon_{cr}$. For graphene substrate, the critical buckling strain is $\varepsilon_{cr}=2.01\%$, which is 27% bigger than the result of atomistic simulation in Section 3, as shown by the solid line in Figure 8. Accordingly, the critical buckling stress $\sigma_{cr}=Et\varepsilon_{cr}$ can be obtained as 5.81 N/m, which is presented by the solid line in Figure 7. The difference between the results of atomistic simulation and theoretical analysis attributes to two main factors: the inaccurate buckling mode and the effect of edge-stress. On the one hand, the trigonometric function wave is not accurate enough to describe the buckling mode of graphene sheet, although it has been widely adopted to study the instability problem of plates. The assumptive mode may be stiffer than the real bulking mode. Thus, under the same uniaxial compressive loading, the theoretical model is more difficult to bulk, resulting in higher critical buckling stress and strain than those of simulation result. On the other hand, the effect of edge stress caused by the unsaturation of atomic bonds along the edges tends to accelerate the buckling process of grapheme [41,42,43], because the reduced bond length along the edges leads to an extra compressive stress [43]. In atomistic simulation, the edge stress is taken into consideration, while it is ignored in the theoretical model. Therefore, in the simulation, the graphene sheet is much easier to buckle, which results in lower critical buckling stress and strain.

Besides, Equations (7) and (8) show that the total energy density *U_T_* and its partial differential $\partial U_{T}/\partial A$ and $\partial U_{T}/\partial \lambda$ are all independent of the graphene size, which indicates that the critical buckling stress and strain are both constants depending only on the substrate material.

**Figure 9:**
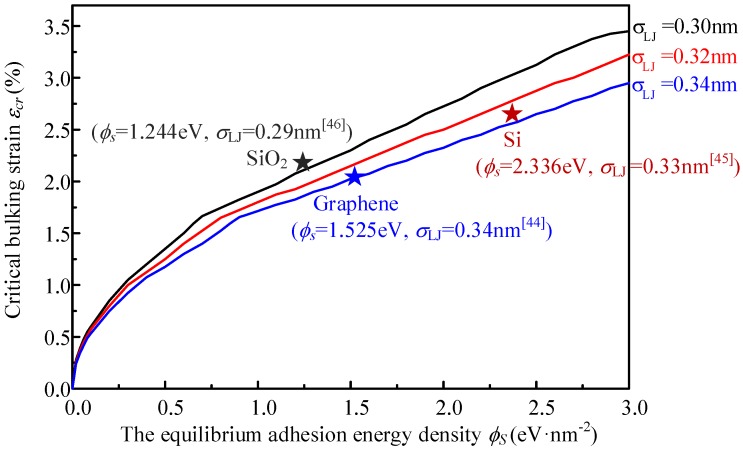
The effect of substrate adhesion on the critical buckling strain. The typical substrate materials are marked by stars.

For different substrate, the adhesion parameters $\sigma_{LJ}$ and $\varepsilon_{LJ}$ and the material density parameter ρ*_s_* are different [44,45,46]. However, in Equation (6), only two independent parameters affect the adhesion energy, *i.e.*, φ*_s_* and $\sigma_{LJ}$, in which φ*_s_* is determined by $\sigma_{LJ}$, $\varepsilon_{LJ}$ and ρ*_s_*. Therefore, the substrate effect can be presented in Figure 9 by the critical buckling strain *versus* φ*_s_*, for substrates with various $\sigma_{LJ}$. The critical buckling strain of some typical substrates, including graphene, Si and SiO_2_ are also presented in Figure 9.

It can be investigated in Figure 9 that the adhesion energy has significant influence on the critical buckling. It is much more difficult to be buckled if the adhesion energy between graphene and substrate is large.

### 3.3. Analytical Approximate Solution

The analytical approximate solution is derived to explicitly present the main factors that influence the critical buckling stress and strain as well as their influence tendency.

In order to attain the analytical solution, the bending energy density *U_b_* in Equation (4) is simplified as:

(9) $$U_{b\;}\approx \frac{1}{2\lambda }\cdot \;\frac{D}{2}\int_{-\lambda }^{\lambda }{\left(\frac{d^{2}u_{z}}{\text{d}x^{2}}\right)}^{2}\text{d}x=\;\frac{DA^{2}\pi^{4}}{4\lambda^{4}}$$

The adhesion energy density can be simplified by Taylor expansion around $u_{z}$ = $\sigma_{LJ}$ as:

(10) $$U_{ad\;}\approx \frac{10\phi_{s}}{\lambda \sigma_{LJ}^{2}}\;\int_{-\lambda }^{\lambda }z^{2}dx=\;\frac{10\phi_{s}A^{2}}{\sigma_{LJ}^{2}}$$

Substituting Equations (9) and (10) into Equations (7) and (8) yields:

(11) $$\lambda^{2}=\;\pi^{2}\sigma_{LJ}\sqrt{\frac{D}{40\phi_{S}}}\;and\;A^{2}=\;-\frac{8D}{Et}+4\sigma_{LJ}\sqrt{\frac{D}{40\phi_{S}}}\cdot \frac{\Delta u_{x}}{l}$$

If A > 0, the graphene sheet buckles, otherwise it keeps flat. Thus, the critical condition can be obtained as:

(12) $$\frac{\Delta u_{x}}{l}=\;\frac{4\sqrt{10D\phi_{s}}}{Et\sigma_{LJ}}$$

Therefore, the critical buckling strain is:

(13) $$\varepsilon_{cr}=\frac{\Delta u_{x}}{l}=\;\frac{4\sqrt{10D\phi_{s}}}{Et\sigma_{LJ}}$$

The critical buckling stress is:

(14) $$\sigma_{cr}=Et\varepsilon_{cr}=\;\frac{4\sqrt{10D\phi_{s}}}{\sigma_{LJ}}$$

For the system with graphene substrate, the critical buckling stress and strain obtained from the analytical approximate solution Equations (13) and (14) are $\varepsilon_{cr}=3.09\%$ and $\sigma_{cr}=8.90\;N/m$, respectively. Although it deviates from the numerical solution because of the linearization of van der Waals energy, the analytical expression can offer a clear relationship between the critical strain and each influence factor. From Equations (13) and (14), it is easy to know that the critical buckling strain $\varepsilon_{cr}$ and critical buckling stress $\sigma_{cr}$ increase nonlinearly with the increasing equilibrium adhesion energy density φ*_s_*, which agrees with the numerical results shown in Figure 9.

## 4. Conclusions

In order to study the compressive buckling behavior of the substrate-supported graphene sheets, both molecular mechanics simulations and energy-based theoretical analysis are carried out to investigate the buckling mode and the critical buckling stress and strain of the monolayer graphene sheets. The following conclusions can be drawn.

(1) The buckling mode of the simple-supported graphene sheet is dominated by the graphene size. For graphene sheets with length larger than 3 nm and width larger than 1.1 nm, the buckling mode depends on the length/width ratio only.
(2) The critical buckling stress and strain of the substrate-supported graphene sheet with two simple-supported edges are about 4.39 N/m and 1.58%, which are dominated only by the substrate adhesion and independent of the graphene size.
(3) The substrate effect is revealed by both theoretical analysis and atomistic simulation. The critical buckling stress and strain can be increased by increasing the adhesive interaction of substrate. The existence of commonly-used substrates can make the critical buckling stress and strain increased by more than 10 times relative to the free-standing graphene sheets.

The estimations on the critical buckling stress and strain obtained in this study can provide guidelines for the design of graphene-derived materials and devices. For example, when the graphene sheet is used as conductive coating materials, the allowable stress of the coated devices should be lower than the critical buckling stress so that the graphene sheet has enough contact area with the substrate to display its high conductivity. Studies have shown that the electrical conductivity of Nano-Electromechanical System and the thermal conductivity of heat conduction element both depend on the smoothness of the graphene sheet [47,48] because the buckling behavior can change the electronic structure of the graphene sheets and make the graphene sheet locally separated from the substrate, which hinders the transfer of electronics, heat and load.

On the other hand, the critical buckling behavior can be utilized to design strain/stress sensors and switches [49] because the electrical conductivity changes significantly with the compressive strain at the critical buckling state.
